# Functions of *SMC2* in the Development of Zebrafish Liver

**DOI:** 10.3390/biomedicines9091240

**Published:** 2021-09-16

**Authors:** Xixi Li, Guili Song, Yasong Zhao, Jing Ren, Qing Li, Zongbin Cui

**Affiliations:** 1 Guangdong Provincial Key Laboratory of Microbial Culture Collection and Application, State Key Laboratory of Applied Microbiology Southern China, Institute of Microbiology, Guangdong Academy of Sciences, Guangzhou 510070, China; lixixi@ihb.ac.cn (X.L.); renjing@ihb.ac.cn (J.R.); 2 Institute of Hydrobiology, Chinese Academy of Sciences, Wuhan 430072, China; guilisong@ihb.ac.cn (G.S.); yszhao@ihb.ac.cn (Y.Z.); qli@ihb.ac.cn (Q.L.); 3 College of Advanced Agricultural Sciences, University of Chinese Academy of Sciences, Beijing 100049, China

**Keywords:** zebrafish, *SMC2*, liver development, DNA damage, apoptosis

## Abstract

*SMC2* (structural maintenance of chromosomes 2) is the core subunit of condensins, which play a central role in chromosome organization and segregation. However, the functions of *SMC2* in embryonic development remain poorly understood, due to the embryonic lethality of homozygous *SMC2^−/−^* mice. Herein, we explored the roles of *SMC2* in the liver development of zebrafish. The depletion of *SMC2*, with the CRISPR/Cas9-dependent gene knockout approach, led to a small liver phenotype. The specification of hepatoblasts was unaffected. Mechanistically, extensive apoptosis occurred in the liver of *SMC2* mutants, which was mainly associated with the activation of the *p53*-dependent apoptotic pathway. Moreover, an aberrant activation of a series of apoptotic pathways in *SMC2* mutants was involved in the defective chromosome segregation and subsequent DNA damage. Therefore, our findings demonstrate that *SMC2* is necessary for zebrafish liver development.

## 1. Introduction

Chromosomes undergo essential changes in morphology, to control the proper expression of genes, and these changes are partially mediated by the structural maintenance of chromosome (*SMC*) proteins [1]. *SMC* proteins are evolutionarily conserved from bacteria to human, and function in chromosome condensation, sister-chromatid cohesion, DNA repair and recombination, and gene dosage compensation in somatic and meiotic cells [2]. *SMC* proteins were initially found in Saccharomyces cerevisiae, and later in all tested eukaryotes [3]. Bacteria contain a single gene that encodes a single *SMC* protein to form homodimers [4]. In eukaryotes, at least six members of the *SMC* protein family are found in individual organisms. The primary structure of *SMC* proteins consists of the following five distinct domains: two nucleotide-binding motifs, Walker A and Walker B motifs that are located in the highly conserved N-terminal and C-terminal domains, respectively, and the central domain, which is composed of a moderately conserved “hinge” sequence that is flanked by two long coiled-coil motifs [5].

As members of the *SMC* family, *SMC2* and *SMC4* form a heterodimer that is the catalytic subunit of both condensins I and II complexes, which play roles in mitotic and meiotic chromosome condensation and rigidity, interphase ribosomal DNA compaction, and removal of cohesion during mitosis and meiosis [6,7,8]. Previous knockout or knockdown studies of *SMC2* revealed its importance for mitotic and meiotic chromosome condensation and segregation in Drosophila melanogaster [9], Caenorhabditis elegans [10], S. cerevisiae [10], and other species [3]. In mice, the knockout of *SMC2* led to embryonic lethality [11]. Thus, the functions of *SMC2* in the embryonic development of vertebrates remain largely unknown.

The liver is a visceral organ in vertebrates that has many important functions in metabolism, secretion, detoxification, and homeostasis. The advantages of high fecundity, transparent embryos, and small size make zebrafish a powerful model for specialized mutagenesis screens, to identify genes whose counterparts can regulate liver development in humans. Liver organogenesis begins with the establishment of a population of cells gaining hepatic competency within the ventral foregut endoderm, instructed by Foxa and Gata factors. Thereafter, mesodermal signals, including Fgfs, Bmps, Wnt2b, and retinoic acid, induce the specification of hepatoblasts, which then migrate and proliferate to form a discrete liver bud. Finally, hepatoblasts in the liver bud undergo rapid proliferation and differentiation, giving rise to bile duct cells and functional hepatocytes [12,13,14,15].

In zebrafish, there are the following three main stages of hepatogenesis: (1) specification (as part of endoderm patterning); (2) differentiation (the budding phase); and (3) hepatic outgrowth, accompanied by morphogenesis [16]. Hepatoblast specification is thought to occur at approximately 22 h post-fertilization, as marked by the localized endodermal expression of *hhex* and *prox1* [14], which are two transcription factor genes that are also expressed in mice [17] and chicks [18]. The budding phase occurs from 24 to 50 hpf. At the subsequent growth stage, the liver undergoes dramatic changes in its size, shape, and placement, because of rapid cell proliferation.

In this study, we addressed an in vivo role of *SMC2* in the liver development of zebrafish. The mutation of *SMC2*, with a CRISPR/Cas9-mediated approach, led to a small liver phenotype, due to elevated apoptosis in the liver and decreased cell proliferation. We then found that extensive apoptosis occurred within the defective liver, due to the activation of intrinsic apoptotic signaling pathways, especially the p53-dependent apoptotic pathway. We further demonstrated that the aberrant activation of the apoptotic pathways was closely associated with DNA damage.

## 2. Materials and Methods

### 2.1. Zebrafish Husbandry and Ethics

Zebrafish AB strain was used and maintained under standard conditions in this study. The *p53^M241K/M214K^* and *Tg(fabp10a:dsRed;ela3l:EGFP)* lines were previously described [19,20]. All zebrafish studies were conducted according to standard animal guidelines and approved by the Institutional Animal Care and Use Committee of the Institute of Hydrobiology, Chinese Academy of Sciences (approval ID: Keshuizhuan 0829).

### 2.2. Generation of SMC2 Mutant Zebrafish Lines

The *SMC2* mutant lines were generated with the CRISPR/Cas9 system following previous methods [21]. Briefly, the target site sequence of *SMC2* is ATCACTGGACTGAACGGCAG, which is located in the second exon. The gRNAs were synthesized in vitro with T7 RNA polymerase (ThermoFisher, Waltham, MA, USA). The Cas9 mRNA was synthesized using the mMESSAGE mMACHINE T7 kit (Invitrogen, Carlsbad, CA, USA). A total of 400 pg Cas9-mRNA and 50 pg *SMC2*-gRNA were co-injected into zebrafish embryos at one-cell stage. The *SMC2* mutations were examined by PCR, and the amplified fragments were sequenced using the following primer pair: 5′-TGGTTGAACTGAAAGCAACG-3′ and 5′-CTTCCAGTTGTTTGCATCTCG-3′.

Because *SMC2^−/−^* died at about 7 days post-fertilization (dpf), the *SMC2^+/c504^ (SMC2^+/−^)* fish were used to cross with *Tg (fabp10a:dsRed; ela3l:EGFP)* fish. To obtain the (*SMC2^+/−^;* DsRed) adult zebrafish, the fluorescence microscope was used to identify DsRed-expressing fish, followed by the genotype of *SMC2^+/−^* fish with the primer pair *SMC2*-gF/*SMC2*-gR (Table S1). The (*SMC2^+/−^;* DsRed) adult zebrafish were inbred to obtain the (*SMC2^−/−^;* DsRed) embryos by phenotypic observation and fluorescence microscope since the *SMC2^−/−^* mutants exhibited abnormal brain and eyes at about 28 h post-fertilization (hpf).

To obtain the (*SMC2^−/−^; p53^−/−^;* DsRed) fish, the (*SMC2^+/−^*; DsRed) adult zebrafish were crossed with *p53^−/−^* fish. Fluorescence microscope was used to identify DsRed-expressing fish, followed by the genotype of *SMC2^+/−^* and *p53^+/−^* with two primer pairs *SMC2*-gF/*SMC2*-gR and tp53-gF/tp53-gR, thus obtaining the (*SMC2^+/−^; p53^+/−^;* DsRed) adult fish. The (*SMC2^+/−^; p53^+/−^;* DsRed) adult fish were inbred to obtain the (*SMC2^−/−^; p53^−/−^;* DsRed) embryos by the observation of dsRed fluorescence and abnormal phenotypes, followed by tail-PCR of 96-hpf embryos with primer pair tp53-gF/tp53-gR (Table S1).

### 2.3. Quantitative Real-Time PCR (qPCR)

Total RNA was extracted from 50 embryos at indicated stages with TRIZOL reagent (Invitrogen, cat#15596026) according to the manufacturer’s instructions. A first strand cDNA synthesis kit (ThermoFisher, Waltham, MA, USA) was used to synthesize cDNAs. SYBR Green real-time PCR master mix (Bio-Rad) was used for qPCR. The qPCR primers used in this study are listed in Table S1.

### 2.4. Whole-Mount In Situ Hybridization (WISH)

Zebrafish embryos at desired stages were fixed in 4% paraformaldehyde (PFA) overnight before processing for WISH analysis as described [22]. Digoxigenin-UTP-labeled antisense RNA probes for *fabp10a*, *cp*, *insulin*, *typsin*, *fabp2*, *hhex*, *prox1*, *foxa1*, *foxa3* and *gata4* were generated with an in vitro transcription method using T7 RNA polymerase (ThermoFisher, Waltham, MA, USA). The primer sequences for these genes are listed in Table S1.

### 2.5. TUNEL Assay

Embryos at 96 hpf were fixed with 4% formaldehyde for 4 h at room temperature and embedded in OCT compound overnight. The embryos were then sectioned at 10 μm thickness using a Leica cryostat. TUNEL assays were performed with in situ cell death detection kit, Fluorescein (Roche, Wilmington, MA, USA), following the manufacture’s instruction.

### 2.6. Immunofluorescence Staining

Sectioned samples were fixed in 4% paraformaldehyde for 20 min and washed five times of 5 min each in PBS. After completely removing paraformaldehyde, samples were permeabilized with 0.5% Triton X-100 (Sigma-Aldrich) in PBS for 15 min at room temperature and blocked in 1% bovine serum albumin for 1 h. Incubation with the primary antibody occurred at 4 °C overnight, followed by overnight incubation with the secondary antibody at 4 °C. Primary antibodies against γ-H2AX (1:400; 9718T; Cell Signaling Technology) were used. The fluorescence-conjugated second antibody FITC goat anti-rabbit IgG (H + L) (BOSTER, Wuhan, China) was applied to sample at a dilution of 1:50. Sections were counterstained with DAPI for 30 min at room temperature and then mounted with an antifade agent. The samples were imaged under an SP8 confocal microscope (Leica).

### 2.7. EdU Labeling

Embryos at 96 hpf were microinjected into the yolk cell with 10 mM EdU. After two hours, the embryos were fixed with 4% formaldehyde for 4 h at room temperature and embedded in OCT compound overnight. The embryos were then sectioned at 10 μm thickness using a Leica cryostat. EdU staining was performed according to the manufacture’s instructions (Beyotime, Shanghai, China).

### 2.8. Synthesis of Capped SMC2 mRNA

The coding sequence of zebrafish *SMC2* was cloned into the vector pSBRNAX and linearized for in vitro transcription. mRNAs were synthesized using the mMESSAGE mMACHINE T7 kit (Invitrogen, Carlsbad, CA, USA). One-cell stage embryos were injected.

### 2.9. Fluorescence-Activated Cell Sorting

Sib and *SMC2^−/−^* mutant embryos at 96 hpf were used. The embryos were transferred into 1.5-mL centrifuge tubes and washed three times in PBS. Then 1 mL of 0.25% trypsin was added to each centrifuge tube. Embryos were passed through the syringe to generate cell suspensions, and the cell suspensions were passed through a 40 μm filter. Cells were washed in ice-cold PBS and incubated for 30 min in 1 mL DAPI. Samples were filtered again over a 40-µm filter, washed with the ice-cold PBS and finally resuspended in PBS for sorting by flow cytometry (BD Biosciences, Franklin Lakes, NJ, USA).

### 2.10. Statistical Analysis

The data are presented as mean ± standard deviation. Statistical differences between two sets of data were analyzed using two-tailed paired Student’s *t*-test, and a value of *p* < 0.05 was considered to indicate significance.

## 3. Results

### 3.1. Conservation of SMC2-Containing Complexes among Vertebrates, and the Expression Patterns of SMC2 during Embryonic Development of Zebrafish

To illuminate the potential conserved functions of *SMC2* among vertebrates, we first compared the similarity of *SMC2*-containing complexes among zebrafish, mice, and humans. The zebrafish genome contains a single copy of each gene encoding subunits of condensin I and II complexes, and the amino acid identity between zebrafish and their human counterparts ranged from 36.5 to 74.2% (Table S2), indicating that the two *SMC2*-containing complexes are highly conserved among vertebrates. Zebrafish *SMC2* encodes a protein consisting of 1199 amino acids. Amino acid sequence alignment indicates that zebrafish *SMC2* is the most conserved subunit, sharing 74.2% identity with human *SMC2* (Table S2).

**Figure 1:**
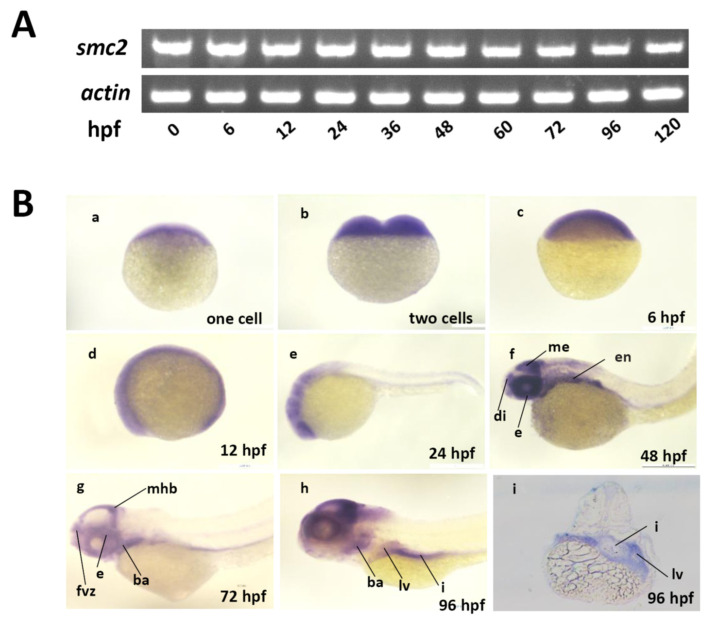
Expression of *SMC2* mRNA during zebrafish embryogenesis. (**A**) Expression levels of *SMC2* at different stages were analyzed with RT-PCR and the expression of *β-actin* served as the control. (**B**) Detection of *SMC2* transcripts during embryogenesis with WISH. hpf, hours post-fertilization; me, mesencephalon; di, diencephalon; fvz, forebrain ventricular zone; ba, branchial arches; mhb, midbrain–hindbrain boundary; i, intestine; en, endoderm; e, eye; lv, liver.

Next, we detected the spatiotemporal expression patterns of *SMC2* with RT-PCR and WISH. Transcripts of *SMC2* can be detected in developing embryos at 0 to 120 hpf (Figure 1A), weakly detected in embryos at the one-cell stage (Figure 1B(a)), and ubiquitously expressed in embryos at two-cells, shield, and 12-hpf stages (Figure 1B(b)–(d)), indicating the maternal origin of *SMC2* transcripts. The *SMC2* transcripts were expressed in diencephalon (di), mesencephalon (me), eye (e), and endoderm (en), at 48 hpf (Figure 1B(f)). Later, its expression was found in the forebrain ventricular zone (fvz), branchial arches (ba), midbrain–hindbrain boundary (mhb), liver (lv), and intestine (i), at 72 and 96 hpf (Figure 1B(g)–(i)). These results indicate that *SMC2* functions in early embryonic development and the formation of multiple organs in zebrafish.

### 3.2. Knockout of SMC2 in Zebrafish

**Figure 2:**
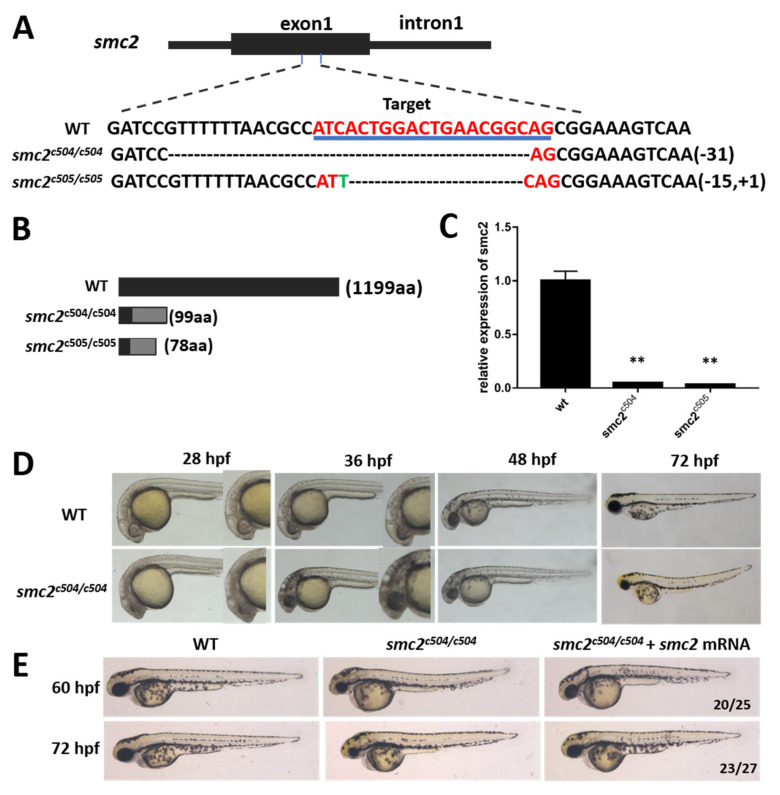
(**A**) Top panel: the schematic diagram shows the structure of the target region in the *SMC2* gene. Black box, exon; solid line, introns. The bottom panel shows a comparison of the genomic DNA sequences among WT and two mutant alleles, *SMC2^c504/504^* mutants carrying a 31-bp deletion and *SMC2^c505/505^* mutants carrying a 15-bp deletion and 1-bp insertion. The target sequence is indicated in red and underlined. (**B**) Schematic structures of WT and truncated *SMC2* proteins. (**C**) The relative mRNA levels of *SMC2* in WT and homozygous mutants were assayed by qPCR at 4 dpf. The results are expressed as the mean ± SD of three independent experiments (**, *p* < 0.01; *t*-test). (**D**) Lateral views showing the morphology of WT and *SMC2^−/−^* mutants at 28, 36, 48 and 72 hpf. (**E**) Morphology of WT and *SMC2^−/−^* embryos injected with or without 200 pg *SMC2*-mRNAs at indicated stages. The smaller eyes and smaller head were significantly reduced in *SMC2*-mRNA-injected mutant embryos. The ratios at the bottom right corners indicate the number of embryos with indicated phenotypes vs. total number of observed embryos.

To investigate the function of *SMC2*, we generated *SMC2* mutant zebrafish with the CRISPR/Cas9 system. Two mutant alleles were obtained from different P0 founders, by targeting exon 1 of the *SMC2* gene. One contains a 31-bp deletion (named *SMC2^c504/c504^*), and the other has a 15-bp deletion and 1-bp insertion (named *SMC2^c505/c505^*) (Figure 2A). Both of the two mutants led to frame-shift mutations of the open reading frame, and premature stop codons that can abolish all functions of *SMC2* (Figure 2B). The relative mRNA expression levels were significantly reduced in homozygotes of *SMC2^c504/c504^* and *SMC2^c505/c505^* embryos (Figure 2C), likely through a nonsense-mediated decay mechanism [23].

Heterozygous *SMC2^c504/+^* or *SMC2^c505/+^* fish that showed no discernable phenotypes are viable, and can develop into fertile adults. However, homozygous mutants obtained from a cross between either *SMC2^c504/c504^* or *SMC2^c505/c505^* homozygotes exhibited a dark phenotype in the head, due to extensive cell death after 28 hpf, and this extended to the whole brain and spinal cord afterwards (Figure 2D). The homozygous mutant embryos displayed small eyes and a small head at 48 and 72 hpf (Figure 2D), and died at about 7 dpf. Since the *SMC2^c504/c504^* and *SMC2^c505/c505^* larvae showed exactly the same phenotypes, *SMC2^c504/c504^* mutants (*SMC2^−/−^* mutants hereafter) were used for further analysis. To determine whether the phenotypes of mutant embryos resulted from *SMC2* depletion, synthesized *SMC2* mRNA was injected into F2 *SMC2^−/−^* larvae. The injection of *SMC2* mRNA did not cause any obvious morphological defects in WT embryos, but markedly rescued the small eyes and small head phenotypes of *SMC2^−/−^* larvae at 60 hpf and 72 hpf (Figure 2E). These findings suggest a crucial role of *SMC2* during zebrafish embryogenesis.

### 3.3. Loss of SMC2 Led to a Small Liver Phenotype in SMC2^−/−^ Mutants

**Figure 3:**
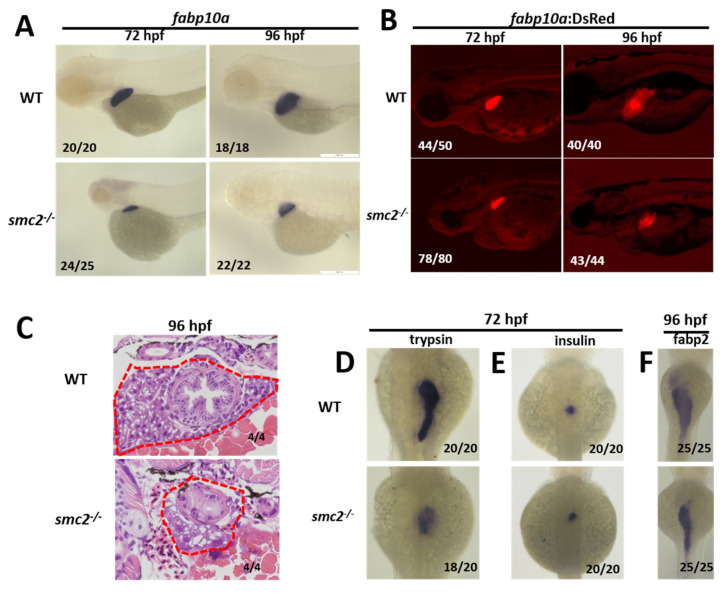
Loss of *SMC2* confers a small liver phenotype. (**A**) WT and *SMC2^−/−^* mutant embryos were stained with RNA probes of *fabp10a*, a marker of hepatocytes at 72 and 96 hpf. (**B**) Liver size in *SMC2^−/−^* mutants is smaller than that in the WT at 72 and 96 hpf, under the *Tg (fabp10a:dsRed; ela3l:EGFP)* transgenic background. (**C**) Livers from WT and *SMC2^−/−^* mutants were analyzed with hematoxylin and eosin staining. (**D**–**F**) WISH using the exocrine pancreas marker *trypsin* (**D**), endocrine pancreas marker *insulin* (**E**), and intestinal maker *fabp2* (**F**) as RNA probes.

The existence of *SMC2* transcripts in the endoderm at 24 hpf, and liver at 96 hpf suggests the involvement of *SMC2* in the liver development of zebrafish. We evaluated liver development in homozygous *SMC2* mutants, using the hepatocyte marker gene *fabp10a* as a probe of WISH. As shown in Figure 3A, the expression of *fabp10a* in *SMC2* mutants at 72 and 96 hpf severely reduced. We also examined the liver phenotype of *SMC2* mutants in the *Tg (fabp10a:dsRed; ela3l:EGFP)* line that expresses DsRed specifically in differentiated hepatocytes. Markedly reduced sizes of livers were shown in *SMC2* mutant larvae in comparison with those in WT larvae, at 72 and 96 hpf (Figure 3B).

Next, the livers in WT and *SMC2^−/−^* mutants were analyzed with hematoxylin and eosin staining, and the small liver in *SMC2^−/−^* mutants at 96 hpf was clearly shown (Figure 3C). The development of an exocrine pancreas and islet was examined by checking the expression of *trypsin* and *insulin* with WISH at 72 hpf, respectively. We found that the exocrine pancreas in *SMC2^−/−^* mutants significantly reduced in size (Figure 3D), while the islet was slightly affected by the loss of *SMC2* (Figure 3E). Moreover, the gut formation was detected with the expression of the intestinal marker gene *fabp2,* and a small gut phenotype was exhibited in *SMC2^−/−^* mutants at 96 hpf (Figure 3F).

Taken together, these data demonstrate that *SMC2* is required for the digestive system development in zebrafish.

### 3.4. SMC2 Is Required for Liver Expansion

**Figure 4:**
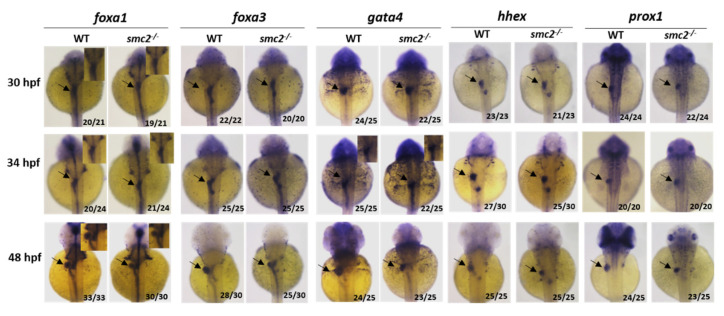
*SMC2* is required for liver expansion. WT and *SMC2^−/−^* mutant embryos were analyzed with markers for liver specification and liver bud expansion at 30, 34 and 48 hpf. WISH probes used include pan-endodermal markers *gata4*, *foxa1*, *foxa3*, and hepatic markers *prox1* and *hhex*. Black arrowhead: liver.

Liver development in zebrafish begins with the specification of hepatoblasts to form a liver bud at about 30 hpf, and these progenitor cells are later expanded and differentiated into either hepatocytes or bile duct cells [24]. We first examined whether the specification of hepatoblasts was affected by the loss of *SMC2*. As shown in Figure 4, the expression patterns of endodermal marker genes, including *foxa1*, *foxa3*, and *gata4*, which are required for the establishment of competent hepatic cells, were similar in the liver *primordioum* of WT and *SMC2^−/−^* mutants at 30 and 34 hpf. We also detected the expression of *prox1* and *hhex*, which are the earliest markers for definitive hepatoblasts [25,26]. The expression of both *prox1* and *hhex* was not affected in *SMC2^−/−^* mutants at 30 and 34 hpf (Figure 4). However, the expression of *foxa1*, *foxa3*, *gata4*, *prox1*, and *hhex*, as well as hepatic marker (*cp*), reduced in the liver region of *SMC2^−/−^* mutants at 48 hpf (Figure 4 and Figure S1). These data indicate that the expansion of the liver bud, but not the specification of hepatoblasts, was affected by the loss of *SMC2*.

### 3.5. Hepatocellular Apoptosis Increased in SMC2^−/−^ Mutants

**Figure 5:**
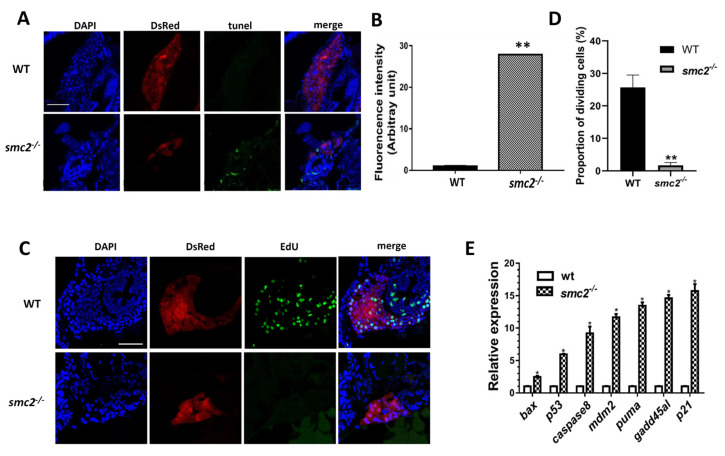
Hepatocellular apoptosis increased in *SMC2*^−/−^ mutants. (**A**) TUNEL analysis of apoptotic cells in the liver of WT and homozygous mutants under the *Tg (fabp10a:dsRed; ela3l: EGFP)* transgenic background at 96 hpf. Scale bar, 50 μm. (**B**) Quantitative analysis of the apoptotic cells in the liver. Fluorescence intensities of three WT and three mutant embryos across the liver were determined using the ImageJ software. **, *p* < 0.01. (**C**) Frozen sections were stained with EdU staining buffer and nuclei counterstained with DAPI (blue) under the *Tg (fabp10a:dsRed; ela3l: EGFP)* transgenic background at 96 hpf. Scale bar, 50 μm. (**D**) The proportions of EdU-positive cells vs. DAPI-positive cells in the liver of three WT and three *SMC2* mutant embryos were determined using the ImageJ software. **, *p* < 0.01. (**E**) The mRNA levels of genes involved in apoptotic pathways were analyzed with qPCR. Expression levels were normalized to WT. The data expressed as mean ± SD were representatives of three independent experiments containing 40 embryos per sample. *, *p* < 0.01.

Since the specification of hepatoblasts was not disturbed in *SMC2^−/−^* mutants, the reduction in liver size may have resulted from increased cell death or a decreased cell proliferation rate [27]. TUNEL assays and EdU (5-ethynyl-2-deoxyuridine) detection were performed in the liver region of zebrafish larvae at 96 hpf. The results of the TUNEL assays indicated that the liver cells of *SMC2^−/−^* mutants underwent active apoptosis, whereas no apoptotic cells were observed in the same region of sibling WT larvae (Figure 5A,B). Moreover, extensive EdU^+^ signals and a high proportion of dividing cells were observed in the liver region of WT larvae, but not in the liver of *SMC2^−/−^* larvae, suggesting that a loss of *SMC2* also reduced the proliferation of liver cells (Figure 5C,D).

We then employed qPCR to detect the expression of a set of genes related to apoptosis during embryogenesis. We found that a number of genes, including *bax*, *p53*, *caspase 8*, *mdm2*, *puma*, *gadd45al*, and *p21*, were highly up-regulated in *SMC2^−/−^* mutants (Figure 5E). These observations indicate that the loss of *SMC2* has triggered intrinsic and extrinsic apoptotic pathways in developing zebrafish.

### 3.6. Activation of the p53-Driven Apoptotic Pathway Contributed to the Small Liver Phenotype in SMC2^−/−^ Mutants

p53 is a key signal molecule of both intrinsic and extrinsic apoptotic pathways, which can regulate the expression of the genes hastening apoptosis and cell cycle arrest [28]. Abnormally elevated expression of *p53* in *SMC2^−/−^* mutants suggests the possibility that p53-dependent apoptosis might be a key cause for the small liver phenotype. To address whether *p53* deficiency could suppress the increased apoptosis in the liver, the *SMC2^+/c504^* (*SMC2^+/−^* hereafter) fish were crossed with *p53^M214K/M214K^* (*p53^−/−^* hereafter) fish [19], and then double heterozygotes (*SMC2^+/c504^; p53^+/M214K^*) were identified by genotyping. Double homozygote mutants (*SMC2^−/−^; p53^−/−^*) were subsequently obtained.

**Figure 6:**
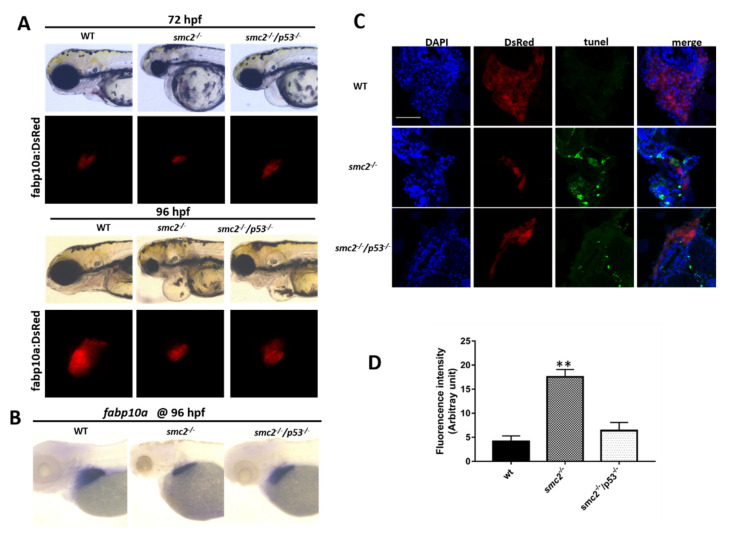
Activation of the p53-dependent apoptotic pathway contributed to the small liver phenotype in *SMC2^−/−^* mutants. (**A**) Phenotype comparison of WT, *SMC2^−/−^* and *SMC2^−/−^/p53^−/−^* embryos under the *Tg (fabp10a:dsRed;ela3l:EGFP)* transgenic background at 72 hpf and 96 hpf. (**B**) WT embryos, *SMC2^−/−^* and *SMC2^−/−^/p53^−/−^* embryos stained with the *fabp10a* probe at 96 hpf. (**C**) TUNEL analysis of apoptotic cells in the liver of *SMC2^−/−^/p53^−/−^* mutants compared to WT and *SMC2^−/−^* mutants at 96 hpf under the *Tg (fabp10a:dsRed;ela3l:EGFP)* transgenic background. Scale bar, 50 μm. (**D**) Quantitative analysis of the apoptotic cells in the liver. Fluorescence intensities of three WT embryos, three *SMC2^−/−^* mutant embryos and three *SMC2^−/−^/p53^−/−^* embryo across the liver were determined using the ImageJ software. **, *p* < 0.01.

We then examined the liver phenotype of the double homozygous mutants that express DsRed from the intercross with the *Tg (fabp10a: dsRed; ela3l: EGFP)* line, and found that the small liver defects of *SMC2^−/−^* mutants at 72 hpf were partially rescued by the loss of p53 (Figure 6A). WISH assays using *fabp10a* as a probe also showed a partially recovered liver in double homozygotes mutants (*SMC2^−/−^; p53^−/−^*) at 96 hpf (Figure 6B). TUNEL assays showed that the number of TUNEL-positive cells in *SMC2* and *p53* double-deficient larvae at 96 hpf was obviously reduced when compared to that in *SMC2^−/−^* mutants (Figure 6C). These data suggest that a loss of p53 could rescue the liver defects, mainly by a reduction in hepatocyte apoptosis in *SMC2^−/−^* mutants.

### 3.7. Extensive Apoptosis Occurring in the Liver of SMC2 Mutants Is Attributable to DNA Damage

**Figure 7:**
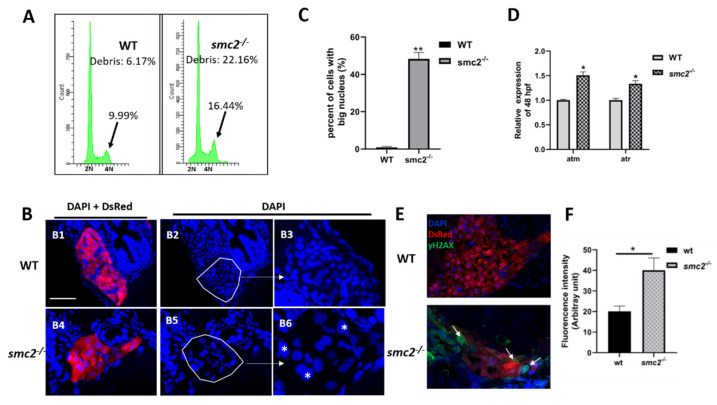
Extensive apoptosis occurring in liver of *SMC2^−/−^* mutants is attributable to DNA damage. (**A**) FACS analyses after DAPI staining of dissociated cells at 96 hpf from wild-type and *SMC2^−/−^* embryos. The *SMC2^−/−^* embryos have an accumulation of cells in the G2/M phase and more debris. (**B**) Frozen sections were stained with DAPI to visualize nuclei under the *Tg (fabp10a:dsRed; ela3l:EGFP)* transgenic background at 96 hpf. The *SMC2^−/−^* embryos have an increased number of cells with bigger nucleus compared with WT. Figure 7(B3,B6) are enlargement of the framed part in Figure 7(B2,B5), respectively. *, bigger nuclei. (**C**) Quantitative analysis of the cell numbers with big nucleus in the liver. Three WT and three mutant embryos across the liver were determined using the ImageJ software. **, *p* < 0.01. (**D**) Expression levels of genes (*atm* and *atr*) implicated in DNA damage response pathway were analyzed by qPCR in WT and *SMC2^−/−^* mutants at 96 hpf. Expression levels were normalized to WT embryos. Data expressed as mean ± SD were representative of three independent experiments. * *p* < 0.01. (**E**) Frozen sections were stained against γ-H2AX antibody, which marks DNA double-stranded breaks and nuclei counterstained with DAPI (blue) under the *Tg (fabp10a:dsRed; ela3l:EGFP)* transgenic background at 96 hpf. The signal was labeled with white arrowhead. (**F**) Quantitative analysis of the apoptotic cells in the liver. Fluorescence intensities of three WT and three *SMC2^−/−^* mutant across the liver were determined using the ImageJ software. **, *p* < 0.01.

It has previously shown that simultaneous depletion of two condensins led to severe defects in chromosome assembly and segregation, which, in turn, caused DNA damage and triggered p53-induced apoptosis in cells [11]. Thus, we suspected that the DNA damage pathway that functions upstream of p53 signaling may be responsible for the small liver phenotype of *SMC2^−/−^* mutants. DNA content analysis with flow cytometry revealed that *SMC2^−/−^* mutants showed an increase, from 9.99% to 16.44%, in mitotic 4N and apoptotic sub-G1 populations (Figure 7A), and a significant increase in the number of cells with a big nucleus (DAPI staining), in comparison with the WT liver (Figure 7B,C). These data demonstrate that a loss of *SMC2* resulted in the blockage of cell cycles at the M-phase, and defects in chromosome segregation.

Defects in chromosome segregation provide a potential source of DNA damage. qPCR was performed to determine the expression levels of *atm* and *atr*, which are the critical components of the DNA damage response pathway. As expected, the qPCR results revealed increased expression levels of these two genes in the DNA damage response pathway in *SMC2* mutants, when compared to those in the WT controls (Figure 7D). Phosphorylated H2AX, referred to as γ-H2AX, can be detected within minutes after the induction of a DNA strand break [29]. Frozen sections were stained with γ-H2AX antibody and nuclei were counterstained with DAPI (blue), under the *Tg (fabp10a:dsRed; ela3l: EGFP)* transgenic background, at 96 hpf. The results revealed an increased number of γ-H2AX foci in the liver of *SMC2^−/−^* mutants when compared to those in the WT controls (Figure 7E,F).

Taken together, these findings indicate that loss of *SMC2* led to defects in chromosome segregation, followed by DNA damage and the activation of apoptotic pathways in *SMC2*-expressing tissues, including the liver region of zebrafish.

## 4. Discussion

Previous studies have shown that condensins play a central role in chromosome organization and segregation [11], but the functions and mechanisms of condensins during the development of the liver in vertebrates remain to be explored. In this study, we demonstrated pivotal roles of zebrafish *SMC2* in the development of the liver. Using the CRISPR/Cas9 technology, we generated two zebrafish homozygous *SMC2* mutant lines, and found that a loss of *SMC2* led to a morphogenetic malformation in the liver. In comparison with WT larvae, homozygous *SMC2* mutants exhibited a small liver phenotype, suggesting a specific function of *SMC2* in the development of the liver. The liver region affected by a loss of *SMC2* matched the position where *SMC2* transcripts were expressed during zebrafish development. Therefore, the tissue-specific expression of *SMC2* in the liver region is required for the appropriate formation of the liver in zebrafish.

The liver morphogenesis process can be arbitrarily divided into the following two phases: budding and growth [13]. The budding phase occurs from 24 to 50 hpf, and at the subsequent growth stage, the liver undergoes dramatic changes in its size, shape, and placement [24]. During the dynamic process, *SMC2* transcripts were detectable in the endoderm and liver region, at and after 48 hpf. A loss of *SMC2* did not impair the specification of hepatoblasts, as characterized by the unperturbed expression of the early endodermal markers *foxa1*, *foxa3,* and *gata4,* as well as the earliest hepatoblast markers *prox1* and *hhex,* at 30 and 34 hpf. However, *SMC2* played indispensable roles in liver expansion, as evidenced by the restricted expression of these makers and *cp* at 48 hpf.

Condensins I and II are two large protein complexes that play a central role in chromosome organization and segregation [30]. Eukaryotic condensins I and II share the core *SMC2* and *SMC4* subunits, but differ in their auxiliary non-*SMC* components, called condensin-associated proteins (CAP-D2, CAP-G, and CAP-H for condensin I; CAP-D3, CAP-G2, and CAP-H2 for condensin II) [31]. Previous studies have shown that condensins I and II are both essential for early embryonic divisions in mice [11,32]. Simultaneous depletion of condensins I and II, from neuronal stem cells, caused severe defects in chromosome assembly and segregation, eventually leading to p53-induced apoptosis [11].

In this study, *SMC2* transcripts were detected in embryos at one-cell stage, indicating its maternal origin. The *SMC2^−/−^* mutant embryos displayed small eyes and small heads at 48 and 72 hpf, and died at about 7 dpf, suggesting that the existence of maternal *SMC2* transcripts would lead to mild mutant phenotypes of *SMC2^−/−^* embryos, and the depletion of maternal *SMC2-*mRNA with *SMC2* morpholinos may cause severe abnormal phenotypes. It is known that a heterodimer of *SMC2* and *SMC4* formed the core of eukaryotic condensins. We found that the abnormal phenotypes of *SMC2^−/−^* mutant embryos can be rescued by the injection of capped *SMC2-*mRNA, and the expression of *SMC4* in embryos at 36 hpf was not upregulated (data not shown). Thus, the mild abnormal phenotypes of *SMC2^−/−^* early developing embryos are caused by the reduction in *SMC2*, but not the redundant function of *SMC4*.

We found that *SMC2* plays critical roles in zebrafish liver morphogenetic processes, since *SMC2* knockout led to an obviously reduced size of the liver. Further evidence from this study indicates that the small liver was caused by increased cell death and reduced cell proliferation in *SMC2^−/−^* mutants. Moreover, increased cell death in the developing liver of *SMC2* mutants was caused by the significantly elevated expression of many genes associated with apoptotic pathways. Among these apoptotic pathways, p53-dependent apoptotic signaling appears to play a key role in the formation of small liver, due to the elevated expression level of *p53* and the partial rescue of the small liver by *p53* knockout in *SMC2* mutants.

p53 is thought to be a decision-making transcription factor that selectively activates genes to determine cellular outcomes [33]. Upon DNA damage, the p53 protein accumulates rapidly through a post-transcriptional mechanism(s), and is also activated as a transcription factor, leading to growth arrest or apoptosis [34,35]. Cells can respond to DNA damage by instigating robust DNA damage response pathways [36], which serve as cellular surveillance systems to sense the presence of damaged DNA, and elicit checkpoint activation and subsequent lesion repair in preventing the amplification or loss of genes or chromosomes [37]. Thus, the activation of p53 apoptotic signaling by the loss of *SMC2* is likely mediated by the abnormality of chromosome organization and segregation. Indeed, we found that *SMC2* mutants had a low fraction of cells in the G1 phase and an accumulation of cells in the G2/M phases. In comparison with WT, *SMC2* mutants exhibited an increased number of cells with a big nucleus, indicating that the knockout of *SMC2* resulted in defects in chromosome segregation. Moreover, we found that defects in the chromosome segregation of *SMC2* mutants led to DNA damage responses, as evidenced by the increased expression levels of *atm* and *atr,* and the significantly elevated expression of γ-H2AX. Thus, the small liver phenotype is mainly attributable to the extensive apoptosis caused by defective chromosome segregation and DNA damage in *SMC2* mutants. 

## 5. Conclusions

The loss of *SMC2* led to a small liver phenotype in *SMC2^−/−^* mutants. The expansion of the liver bud, but not the specification of hepatoblasts, was affected by the loss of *SMC2*. Increased cell apoptosis and decreased cell proliferation are responsible for the small liver phenotype in *SMC2^−/−^* mutants. The p53-driven apoptotic pathway was activated in *SMC2^−/−^* mutants. Extensive apoptosis occurring in the liver of *SMC2* mutants is attributable to DNA damage.

## Data Availability

Data are contained within the article.

## Supplementary Materials

The following are available online at https://www.mdpi.com/article/10.3390/biomedicines9091240/s1, Figure S1: WISH of specific maker (*cp*) for hepatic cells; Table S1: the primers used in this study; Table S2: comparison of condensing I and condensing II subunits of human, mouse, and zebrafish.
