# Supplementary material for: Functions of SMC2 in the Development of Zebrafish Liver
Source: Biomedicines. 2021 Sep 16;9(9):1240. doi: 10.3390/biomedicines9091240 (PMC8465584; doi:10.3390/biomedicines9091240)
Supplement: Supplementary file 1 [file biomedicines-09-01240-s001.zip › biomedicines-1360272-supplementary.pdf]

Table S1. The primers used in this study

qPCR Primers	Sequences (5′-3′)
smc2-F	TAAAGTCGATTGTACTAGAGGGCTTC
smc2-R	GGAGATTTGTGGCTCGAACCT
atr-F	ACCAGGCAGTGCAGAAACCTC
atr-R	ATGTGCTGCACAAAGTCTAACAGC
atm-F	TGCACGAGCTGTTAGTGTGCTG
atm-R	AGCTGTTTGGAGCCTTTGCTTC
caspase8-F	GGAGAGAGAAAGGAGGAGAAAC
caspase8-R	CCGCTGGGTCAGTATGTAAT
bbc3-F	GGTTTCAAGCACTTCCCTTAGA
bbc3-R	CCGACGCAAACACAGAAATG
gadd45al-F	GACGGAAGCTCCTTCAGAATAC
gadd45al-R	GTCCTCAGAAAGTCCCACAAA
mdm2-F	AACAGCAACTCGGATGTAGG
mdm2-R	CCACCTCAAACTCCACACTAA
tp53-F	GTACAAGTCCCTCCTGGAAATC
tp53-R	GGCAAATGCGTGTAAACAGTAA
**Genotype primers**	**Sequences (5′-3′)**
smc2-gF	TGGTTGAACTGAAAGCAACG
smc2-gR	CTTCCAGTTGTTTGCATCTCG
tp53-gF	TGCCAGAGTATGTGTCTGTC
tp53-gR	TGAGAGCAGCATCATGAAGC
**WISH primers**	**Sequences (5′-3′)**
smc2-pF	AAGGTGAACTGAGGCGAAG
smc2-pR	TGAGCATCGTTGTTTTGCTC
fapb10-pF	AGCTTCTCCAGAAAGCATGG
fapb10-pR	AGTGATGGTGAAACGCTTCAG
fabp2-pF	TCATCATCATGACCTTCAACG
fabp2-pR	TCACAGGTGCAAATGACACG
trypsin-pF	ACACCACTCAACAGCTTCACC
trypsin-pR	AGATGGTATTGCAACACGCC
insulin-pF	CCATATCCACCATTCCTCGC
insulin-pR	CAAACGGAGAGCATTAAGGC
cp-pF	AAACCCTCCCAAAGCACTGAC
cp-pR	TACCCACTCCCGAATATGCC
foxa1-pF	TGGATTTCCTCCATAATGTTGGG
foxa1-pR	AAGGGTCCATGCTGGCTCTC
foxa3-pF	TGAGGCAAATGAGATGTATTCCTC
foxa3-pR	TAGCTGTTGTAGGCCATCACCT
gata4-pF	GATATGTATCAAGGTGTAACGATGG
gata4-pR	TGGTGTGTGTAGAGCGGTGT
hhex-pF	TCCATCATGCAATTCCAGCAC
hhex-pR	CATAGGGTGAACTGATGCTCGT
prox1-pF	GAGGCAGTGAGCCTAATTTC
prox1-pR	CAGAGAGGGATGGTGGTGGT

Table S2. Comparison of condensing I and condensing II subunits of human, mouse and zebrafish

	Human Genes	Human Proteins (AA NO.)	Mouse orthologs (AA NO. / Identities)	Zebrafish orthologs (AA NO. / Identities)
Condensin I-Specific subunits	NCAPD2/CAP-D2	NCAPD2(1401)	1392/85.9	1380/48.5
	NCAPG/CAPG	NCAPG(1015)	1004/82	1003/50.2
	NCAPH/CAP-H	NCAPH(741)	731/77.5	690/46
Shared subunits	SMC2/CAP-E	SMC2(1197)	1191/91.6	1199/74.2
	SMC4/CAP-C	SMC4(1288)	1286/89.5	1289/68.2
Condendin II-specific subunits	NCAPD3/CAP-D3	NCAPD3(1498)	1506/72.7	1419/43
	NCAPG2/CAP-G2	NCAPG2(1143)	1138/81	1140/45.1
	NCAPH2/CAPH2	NCAPH2(606)	607/78.4	592/36.5

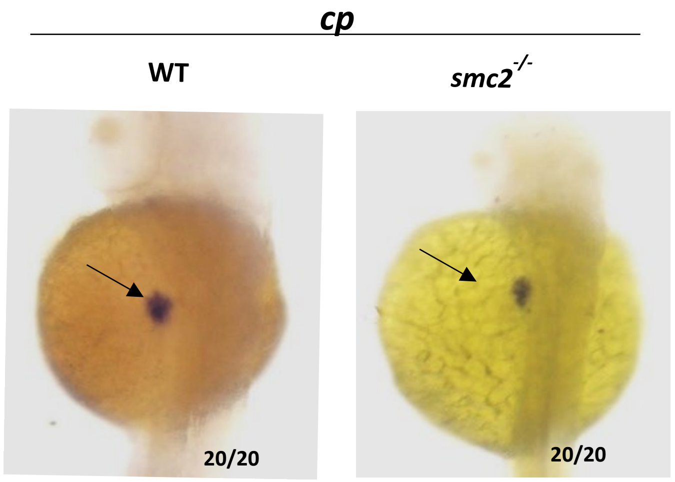

Figure S1. WISH of specific maker (*cp*) for hepatic cells.

WT and smc2$^{-/-}$ mutant embryos were stained with RNA probes of cp, a marker of hepatocytes at 48 hpf.
